# Trends and determinants of adolescent childbirth in Uganda- analysis of rural and urban women using six demographic and health surveys, 1988–2016

**DOI:** 10.1186/s12978-020-00925-8

**Published:** 2020-05-26

**Authors:** Dinah Amongin, Lenka Benova, Annettee Nakimuli, Mary Nakafeero, Frank Kaharuza, Lynn Atuyambe, Claudia Hanson

**Affiliations:** 1 Department of Obstetrics and Gynaecology, School of Medicine, Makerere University College of Health Sciences, Kampala, Uganda; 2 Department of Health Policy Planning and Management, Makerere university School of Public Health, Kampala, Uganda; 3 Faculty of Epidemiology and Population Health, London School of Hygiene and Tropical Medicine, London, UK; 4 Department of Public Health, Institute of Tropical Medicine, Antwerp, Belgium; 5 Department of Epidemiology and Biostatistics, Makerere University School of Public Health, Kampala, Uganda; 6 Department of Community Health and Behavioral Sciences, Makerere University School of Public Health, Kampala, Uganda; 7 Department of Disease Control, London School of Hygiene & Tropical Medicine, London, UK; 8 Dept of Global Public Health, Karolinska Institutet, Solna, Sweden

**Keywords:** Trends, Determinants, Adolescent, Childbirth, Pregnancy, Rural, Urban, Uganda, Surveys

## Abstract

**Introduction:**

Uganda has high adolescent pregnancy. The details of adolescent childbirth and urban/rural patterns are scarce. We investigated the levels, time trends and determinants of adolescent childbirth in Uganda separately for urban and rural women.

**Methods:**

We estimated the percentage of women 20–24 years at each of the six Uganda Demographic and Health Surveys (1988/89, 1995, 2000/01, 2006, 2011 and 2016) who reported a live childbirth before age 20 years (“adolescent childbirth”), and examined change over time using t-test. A modified multivariable Poisson regression was used to examine determinants of having adolescent childbirth on the 2016 survey.

**Results:**

Among these women, 67.5, 66.4, 70.1, 62.3, 57.3 and 54.1% reported an adolescent childbirth in 1988/89, 1995, 2000/01, 2006, 2011 and 2016 surveys, respectively. Between 1988/89 to 2000/01, there was no evidence of change (+ 2.6% point (pp), *p* = 0.170), unlike between the 2000/01 and 2016 surveys when a significant decline occurred (− 16.0 pp., *p* < 0.001). First childbirth < 18 years of age declined by − 13.5 pp. (p < 0.001) between 2000/01 and 2016. There was no change over time in the percentage of adolescents 18–19.9 years of age having first childbirth. Among rural residents, childbirth < 18 years declined from 43.8% in 1988/89 to 32.7% in 2016 (− 11.1 pp., p < 0.001), in urban it declined from 28.3 to 18.2% (− 10.1 pp., *p* = 0.006). There was an increase over time in the percentage of women, both rural and urban, who wanted to delay their first pregnancy. Independent determinants of reporting an adolescent childbirth in both urban and rural residents were: no education/incomplete primary and younger age at first sex. Additional determinants for rural women were residence in Eastern region, Muslim religion, and poor household wealth index.

**Conclusion:**

In the 30-year period examined, adolescent childbirth in Uganda declined from highs of 7 in 10 to approximately 5 in 10 women, with more wanting to delay the pregnancy. The decline started after the 2000/01 survey and affected predominantly younger adolescent childbirth < 18 years among both rural and urban residence women. Efforts need to be intensified to sustain the decline in adolescent pregnancies. Targeted and specific strategies for urban and rural areas might be required.

## Plain English summary

In Uganda, it is not clear how many adolescent girls give birth over the years among both rural and urban residents. Further, it is not clear which girls are more likely to give birth before 20 years. To answer this, we used information among women 20–24 years of age from the 6 government surveys; the Uganda Demographic and Health Survey that capture information on the health, 1988/89 to 2016.

We found that 7 in 10 young women reported having had a live baby and the number reduced to 5 in 10 by the time of the 2016 survey. The number started reducing after the 2000/01 survey. The reductions occurred in women having a live baby before 18 years of age but not among those between 18 and 19 years of age. Similar changes occurred among women residing in both rural and urban areas. Over the years, more women wanted to delay the pregnancy. At 2016 survey, the girls who were more likely to report a birth, in both residences, were those with no education/incomplete primary education and those who begun sex at a younger age. Rural Muslim girls and the poor were more at risk.

In conclusion, live birth before 20 years of age reduced after 2000 survey, among birth rural and urban women.. Government in Uganda needs to prevent pregnancy while considering the differences between girls from rural and urban areas; the Muslim families and what helped reduce births among the girls < 18 years of age.

## Introduction

Globally, approximately 17 million adolescent births occur annually and 95% of these occur in low- and middle-income (LMIC) countries [1–3]. Adolescent childbirth is associated with adverse health, social and economic outcomes to the girl and her offspring [2, 4, 5]. The adolescent girls from poorer settings are at higher risk of adverse outcomes [3, 6]. The poor outcomes can be immediate or continue into later life [7–10]. The risk increases with younger age and those below 18 years of age are most vulnerable due to their premature physiologic development [5, 11, 12]. Sub-Saharan Africa has the highest proportion of adolescents - 23% of its population - and bears 50% of the global adolescent births’ burden [2, 3, 13–15].

In Uganda, the 2016 Uganda Demographic and Health Survey (UDHS) indicates that approximately 25% of girls age 15–19 years had begun childbearing (were either pregnant or had given birth at time of the survey) and this percentage is similar to 2011 and 2006 UDHS [16–18]. Information on the proportions of adolescents who give birth, over the entire period of adolescence (10–19 years) is not clear. The available estimates cover 15–19-year olds.

A number of interventions are instituted to prevent adolescent pregnancies and childbirths especially among the girls < 18 years of age with varying success in sub-Sahara Africa [19–21]. In Uganda, government strategies to address this problem include introduction of universal primary education (UPE) and universal secondary education (USE), contraception for adolescents, enactment of the defilement law and prohibition of marriage for girls < 18 years of age [22–24]. These strategies were introduced around the period of the 2000/01 survey and have been intensified in the last 15 years. There are limited data regarding whether the adolescent childbirth is changing during this period and in respect to the age cut-offs for defilement and legal marriage. It is also not known whether the changes, if any, are among both rural and/or urban adolescents. Urban and rural settings, Uganda inclusive, are not homogenous- there may be differences including in: adolescent access to educational opportunities, household wealth, contraception information and services, and other social amenities [25–28] that may have influenced adolescent childbirth.

This study examined the levels and trends in adolescent childbirth (childbirth < 20 years) among rural and urban women using UDHS data from the last six surveys while disaggregating by age cut offs in view of the policies; < 18 years and 18–19.9 years. We discussed the findings in light of the period when interventions were introduced. Further, we examined whether this first adolescent childbirth was intended (=wantedness) and the proportions of women who were in union before this childbirth. The determinants of adolescent childbirth among women of rural and urban residence were identified using the 2016 UDHS data, in order to improve implementation of these adolescent childbirth prevention strategies.

## Methods

### Study design and data sources

This study utilized data from the UDHS rounds of 1988/89, 1995, 2000/01, 2006, 2011, and 2016. These multistage nationally representative surveys of households are conducted in Uganda every five years and collect information on population health along with the socioeconomic and demographic characteristics of respondents. All women aged 15–49 years in sampled households provide self-reported information in the individual woman’s questionnaire about all the live births they have had. The 1988/89 and 2000/01 surveys excluded districts that accounted for 20 and 5% of the national population, respectively, due to security concerns mainly in northern Uganda. The results presented for these two surveys are representative of the non-excluded districts only.

### Study variables

We utilized data for women in the age category 20–24 years at the time of each survey. Our main outcome was adolescent childbirth defined as a live childbirth at < 20 years of age and disaggregated into < 18 years and 18.0–19.9 years. The category of women age 20–24 years was chosen due to completion of the period at risk for adolescent childbirth. Over the six consecutive surveys, there were some changes to the questionnaire, but in this study we used only variables that were consistently collected in all the surveys, except the question on whether the pregnancy was wanted then, later or no more (pregnancy wantedness), which was introduced in the 1995 survey. The determinants examined include socio-demographic (zones, residence, religion, household wealth quintile, education attainment, being in union before first birth, and age at first sex). Regions were re-categorized into four geographic zones based on the categorization in the 2000/01 survey: Central, South, North and East, in order to harmonise geographical areas across all six surveys due to changes in regional boundaries over time, as done in other studies [29]. Central zone contained Kampala, Central 1 (South Buganda) and Central 2 (North Buganda). Northern zone included Lango, Acholi and West Nile sub-regions. Eastern zone was composed of Teso, Karamoja, Bugisu, Bukedi, and Busoga sub-regions while Western zone was composed of Bunyoro, Tooro, Ankole and Kigezi sub-regions. Education attainment was collapsed into two categories: 1) no education and incomplete primary and 2) complete primary and higher levels of education. Union (married or living together) at first childbirth was categorized into two: union before first childbirth and no union before first childbirth (includes those reporting union and childbirth took place at the same age in years). Religion was re-categorized into four categories; Anglican (Anglican and Pentecostal/born again/evangelical), Catholic, Muslim, and other (Seventh day Adventist, orthodox, Baptist, traditional, no religion, other, and Jehovah’s Witness). Residence (urban or rural), household wealth quintile, and age at first sex were used as captured in the survey datasets.

### Data management and analysis

We calculated the percentage of women with the outcome (and associated 95% confidence interval) and the percentage point (pp) change in the outcome from between the surveys. Wantedness and proportion in union before first childbirth were calculated on surveys starting with 1995 as the question on wantedness of the pregnancy was introduced on the 1995 survey. The two-sample test of proportions was used to obtain the *p*-value of the difference. We calculated outcomes stratifying between the rural and urban residence. Using the most recent 2016 survey data, we conducted three levels of analysis: descriptive, bivariate and multivariable analysis for rural and urban residence women separately. There were no missing values in the variables used. Modified Poisson regression was used for crude and multivariable analysis because the prevalence of the outcome was common, above 10%. In cross sectional studies, when the prevalence of outcome is common, there are alternatives to the logistic regression such as modified Poisson regression, to avoid over estimating the prevalence ratios [30]. All factors were included in the multivariate analysis irrespective of the crude association *p*-value. Alpha was set at 5 and 95% confidence interval. Analysis was corrected for sampling design using population weights, clustering and stratification adjustments. STATA version 12.0, StataCorp LP, Texas was used for the analysis.

## Results

### Levels and time trends in adolescent childbirth

We analysed data for women aged 20–24 years from all six surveys. The total sample was: 985 in 1988/89, 1555 in 1995, 1504 in 2000/01, 1710 in 2006, 1629 in 2011, and 3822 in 2016 *(*Table 1*).* Over 70% of the women at each survey point were from rural residence and this proportion declined from 86.7% in 1988/89 to 70.1% in 2016 (− 16.6 percentage points (pp), *p* < 0.001) in the 30 years. Among all women, 67.5, 66.4, 70.1, 62.3, 57.3 and 54.1% reported an adolescent childbirth in 1988/89, 1995, 2000/01, 2006, 2011 and 2016 surveys, respectively. When this was disaggregated by age category, 25.8% of all women, in 1988/89 reported first adolescent birth between 18 and 19.9 years, compared to 28.2 and 25.8% in 2000/01 and 2016 respectively. Those reporting adolescent birth at < 18 years were 41.7% in 1988/89 compared to 41.9% in 2000/01 and 28.4% in 2016.

**Table 1:** Trends in residence and adolescent childbirth among Ugandan women age 20–24 years, UDHS 1988/9–2016

UDHS (Data collection)						
**Category**	**1988/89**	**1995**	**2000/01**	**2006**	**2011**	**2016**
	***N*** **= 985%(95%CI)**	***N*** **= 1555%(95%CI)**	***N*** **= 1504%(95%CI)**	***N*** **= 1710%(95%CI)**	***N*** **= 1629%(95%CI)**	***N*** **= 3822%(95%CI)**
**Rural residence**	86.7	84.1	80.2	78.0	74.5	70.1
	(84.3–88.8)	(81.3–86.5)	(76.9–83.1)	(74.6–81.1)	(70.6–78.0)	(67.0–73.0)
**Urban residence**	13.3	15.9	19.8	22.0	25.5	29.9
	(11.2–15.7)	(13.5–18.7)	(16.9–23.1)	(18.9–25.4)	(22.0–29.4)	(27.0–33.0)
**No birth < 20 yrs**	32.5	33.6	29.9	37.7	42.7	45.9
	(29.0–36.2)	(30.9–36.4)	(26.9–33.1)	(34.8–40.7)	(39.4–46.2)	(43.8–48.0)
**1st birth 18–19.9 yrs**	25.8	27.3	28.2	27.0	24.2	25.8
	(23.3–28.5)	(24.5–30.3)	(25.8–30.7)	(24.6–29.6)	(21.9–26.7)	(24.1–27.5)
**1st birth < 18 years**	41.7	39.1	41.9	35.2	33.0	28.4
	(38.0–45.5)	(36.0–42.3)	(38.6–45.4)	(32.7–37.9)	(30.1–36.1)	(26.6–30.2)
**Percent-point changes (pp) in adolescent childbirth by intervals (*p*-value)**						
**Intervals**	**1988/89–1995**	**1995–2000/01**	**2000/01–2006**	**2006–2011**	**2011–2016**	**1988–2000/01**
**No birth < 20 years**	+ 1.1 (0.566)	−3.7 (0.028)	+ 7.8 (< 0.001)	+ 5.0 (0.003)	+ 3.2 (0.030)	− 2.6 (0.170)
**1st birth 18–19.9 years**	+ 1.5 (0.405)	+ 0.9 (0.578)	−1.2 (0.447)	− 2.8 (0.064)	+ 1.6 (0.214)	+ 2.4 (0.189)
**1st birth < 18 years**	− 2.6 (0.193)	+ 2.8 (0.115)	−6.7 (< 0.001)	−2.2 (0.180)	−4.6 (< 0.001)	+ 0.2 (0.921)
			**2000/01–2016**			**1988/89–2016**
**No birth < 20 years**			+ 16.0 (< 0.001)			+ 13.4 (< 0.001)
**1st birth 18–19.9 years**			−2.4 (0.074)			0.0 (1.000)
**1st birth < 18 years**			−13.5 (< 0.001)			−13.3 (< 0.001)

Between the 1988/89 and 2000/01 surveys, there was no evidence of a decline in adolescent childbirth overall or in either age category of first childbirth (Table 1.). During this period, the percentage of women reporting first birth between 18 and 19.9 years changed by + 2.4 pp. (*p* = 0.189) whereas in those < 18 years, it changed by + 0.2 pp. (*p* = 0.921). An evidence of a decline in adolescent birth was observed between 2000/01 and 2016 among the age category of < 18 years (− 13.5 pp., *p* < 0.001), but not among those between 18 and 19.9 years (− 2.4 pp., *p* = 0.074).

Among rural residents, a pattern of decline in adolescent childbirth similar to national trends was observed. The proportion of women reporting adolescent childbirth among these women was 69.9% in 1988/89, 74.8% at 2000/01 survey and thereafter declined to 60.6% at the 2016 survey (−9.3 pp., p < 0.001), as shown in Table 2. This change was due to first childbirth < 18 years which declined after 2000/01 survey, from 45.0% in 2000/01 to 32.7% in 2016 (− 12.3 pp., *p* < 0.001). There was no evidence of a change in the percentage or rural women reporting first childbirth between 18 and 19.9 years in the entire period of observation: 26.1% in 1988/89 to 27.9% in 2016 (+ 1.8 pp., *p* = 0.305).

**Table 2:** Levels and percent-point changes in adolescent childbirth among Ugandan women aged 20–24 - RURAL residents

UDHS (Data collection)						
	**1988/89*N*** **= 854**%(95%CI)	**1995*N*** **= 1307**%(95%CI)	**2000/01*N*** **= 1206**%(95%CI)	**2006*N*** **= 1334**%(95%CI)	**2011*N*** **= 1213**%(95%CI)	**2016*N*** **= 2678**%(95%CI)
**No birth < 20 years**	30.1	31.1	25.2	31.9	36.4	39.4
	(26.4–34.1)	(28.0–34.3)	(21.7–28.9)	(29.0–35.0)	(32.5–40.4)	(37.1–41.8)
**1st birth 18–19.9 years**	26.1	27.1	29.9	29.4	27.4	27.9
	(23.3–29.1)	(23.9–30.5)	(27.0–32.8)	(26.5–32.4)	(24.7–30.2)	(26.0–29.8)
**1st birth < 18 years**	43.8	41.8	45.0	38.7	36.2	32.7
	(39.7–48.0)	(38.2–45.5)	(41.0–49.0)	(35.8–41.6)	(32.6–40.0)	(30.5–34.9)
**Percent-point changes (pp) in adolescent childbirth by intervals (*p*-value)**						
**Intervals**	**1988/89–1995**	**1995–2000/01**	**2000/01–2006**	**2006–2011**	**2011–2016**	**1988–2000/01**
**No birth < 20 years**	+ 1.0 (0.622)	−5.9 (0.001)	+ 6.7 (< 0.001)	+ 4.5 (0.017)	+ 3.0 (0.075)	−4.9 (0.014)
**1st birth 18–19.9 years**	+ 1.0 (0.608)	+ 2.8 (0.120)	−0.5 (0.783)	−2.0 (0.264)	+ 0.5 (0.747)	+ 3.8 (0.059)
**1st birth < 18 years**	− 2.0 (0.358)	+ 3.2 (0.106)	−6.3 (0.001)	− 2.5 (0.193)	−3.5 (0.033)	+ 1.2 (0.589)
			**2000/01–2016**			**1988/89–2016**
**No birth < 20 years**			+ 14.2 (< 0.001)			+ 9.3 (< 0.001)
**1st birth 18–19.9 years**			−2.0 (0.202)			+ 1.8 (0.305)
**1st birth < 18 years**			−12.3 (< 0.001)			−11.1 (< 0.001)

Among urban women, adolescent childbirth declined from 51.9% in 1988/89, 50.9% in 2000/01 to 39.0% in 2016 (−12.9 pp. difference, *p* = 0.004) (Table 3). The percentage of women reporting first childbirth < 18 years registered a decline after the 2000/01 survey, from 29.5% in 2000/01 to 18.2% in 2016 (− 11.3 pp. difference, *p* < 0.001). There was no evidence of a decline in first adolescent birth between 18 and 19.9 years in the entire period of observation, 23.6% in 1988/89 and 20.8% in 2016 (− 2.8 pp., *p* = 0.457).

**Table 3:** Levels and percent-point changes in adolescent childbirth among Ugandan women aged 20–24 - URBAN residents

	UDHS (data collection)					
	**1988/89*N*** **= 131**%(95%CI)	**1995*N*** **= 248**%(95%CI)	**2000/01*N*** **= 298**%(95%CI)	**2006*N*** **= 376**%(95%CI)	**2011*N*** **= 416**%(95%CI)	**2016*N*** **= 1144**%(95%CI)
**No birth < 20 years**	48.1	46.8	49.1	58.3	61.2	61.0
	(39.7–56.5)	(41.9–51.7)	(44.3–54.0)	(51.6–64.8)	(55.8–66.3)	(56.8–65.0)
**1st birth 18–19.9 years**	23.6	28.6	21.3	18.7	15.1	20.8
	(19.3–28.6)	(24.6–32.9)	(17.7–25.5)	(14.1–24.3)	(11.1–20.2)	(17.7–24.3)
**1st birth < 18 years**	28.3	24.6	29.5	23.0	23.7	18.2
	(21.8–35.9)	(20.6–29.1)	(25.1–34.4)	(18.7–27.9)	(20.0–27.9)	(15.7–21.0)
**Percent-point changes (pp) in adolescent childbirth by intervals (*p*-value)**						
**Intervals**	**1988/89–1995**	**1995–2000/01**	**2000/01–2006**	**2006–2011**	**2011–2016**	**1988–2000/01**
**No birth < 20 years**	−1.3 (0.809)	+ 2.3 (0.592)	+ 9.2 (0.017)	+ 2.9 (0.406)	−0.2 (0.943)	+ 1.0 (0.849)
**1st birth 18–19.9 years**	+ 5.0 (0.296)	−7.3 (0.049)	−2.6 (0.401)	−3.6 (0.176)	+ 5.7 (0.012)	− 2.3 (0.596)
**1st birth < 18 years**	− 3.7 (0.434)	+ 4.9 (0.201)	−6.5 (0.056)	+ 0.7 (0.816)	−5.5 (0.016)	+ 1.2 (0.801)
			**2000/01–2016**			**1988/89–2016**
**No birth < 20 years**			+ 11.9 (< 0.001)			+ 12.9 (0.004)
**1st birth 18–19.9 years**			−0.5 (0.850)			−2.8 (0.457)
**1st birth < 18 years**			−11.3 (< 0.001)			−10.1 (0.006)

**Fig. 1:**
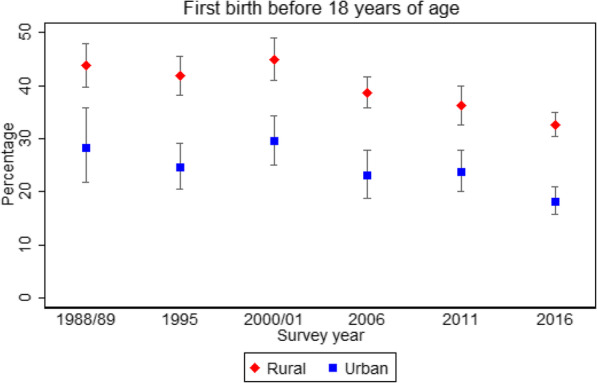
Trends in adolescent childbirth < 18 years among rural and urban Ugandan women aged 20–24, all UDHS surveys

Compared to women of rural residence, those of urban residence had lower proprotions reporting a first adolescent birth at each point of survey (Tables 2 and 3). A statistical evidence of a decline occurred after the 2000/01 survey among both categories and this was due to decline in first birth < 18 years (Fig. 1). The 1988/9 levels of first birth < 18 years were higher among rural women compared to urban women (43.8% compared to 28.3%,) and by 2016 both declined by a similar absolute point change (− 11.1 pp. rural, − 10.1 pp. urban).

### Wantedness of pregnancy and marital status at the first adolescent birth, 1995 to 2016 UDHS surveys

Regarding wantedness of the pregnancy for the first birth, there was a decline in the percentage of women who reported having wanted the pregnancy at the time, among both rural and urban residents (Table 4). The decline among both categories was − 19.5 pp. (*p* < 0.001) from 1995 to 2016. Among rural residents, those who wanted the pregnancy then were: 80.7% in 1995 and 63.0% in 2016 (decline of − 17.7 pp., p < 0.001). For the urban residents, those who wanted the pregnancy at the time declined from 74.6% in 1995 to 50.2% in 2016 (− 24.4 pp., p < 0.001).

**Table 4:** Wantedness of and marital status at first adolesecent birth among Uganda women age 20–24 years, 1995–2016 UDHS

	Survey year				
**Total Sample with adol. Birth (< 20 years)**	**1995**	**2000/01**	**2006**	**2011**	**2016**
	***N*** **= 1033**	**N = 1504**	***N*** **= 1065**	***N*** **= 933**	***N*** **= 2068**
**In Union/marriage before first birth in adolescence (column %; 95% CI)**					
**All women**	64.9	67.3	64.5	56.8	55.8
	(61.3–68.4)	(63.6–70.7)	(60.8–68.1)	(52.8–60.8)	(53.1–58.5)
**Rural women**	**901**	**903**	**908**	**771**	**1622**
	66.4	69.2	66.0	58.7	56.4
	(62.3–70.2)	(65.2–73.0)	(62.0–69.8)	(53.9–63.2)	(53.5–59.3)
**Urban women**	**132**	**152**	**156**	**162**	**446**
	54.9	55.5	55.7	48.2	53.7
	(49.3–60.5)	(48.8–61.9)	(45.5–65.4)	(41.4–55.0)	(47.1–60.1)
**N analyzed for wantedness (all with birth < 20 years), % and 95 CI**					
**All women with record**	**354**	**545**	**570**	**490**	**1111**
**Then**	79.7	76.6	65.7	64.4	60.2
	(74.0–84.4)	(71.7–80.9)	(61.0–70.0)	(59.3–69.2)	(56.6–63.8)
**Later**	19.8	14.5	29.3	33.7	37.7
	(15.2–25.5)	(11.0–18.8)	(25.0–34.0)	(28.8–39.0)	(34.2–41.3)
**No more**	0.5	8.9	5.0	1.9	2.1
	(0.1–2.3)	(6.3–12.4)	(3.3–7.5)	(0.9–3.8)	(1.1–3.9)
**Rural women**	**298**	**466**	**485**	**407**	**872**
**Then**	80.7	79.4	65.6	64.9	63.0
	(74.0–85.9)	(73.9–84.0)	(60.4–70.5)	(59.0–70.3)	(59.0–66.8)
**Later**	18.9	14.0	30.2	33.8	36.2
	(13.8–25.4)	(10.2–19.0)	(25.4–35.4)	(28.4–39.7)	(32.5–40.2)
**No more**	0.4	6.6	4.2	1.3	0.8
	(0.1–3.1)	(4.0–10.7)	(2.5–6.9)	(0.5–3.1)	(0.4–1.8)
**Urban women**	**056**	**079**	**085**	**083**	**239**
**Then**	74.6	60.1	66.1	62.1	50.2
	(62.6–83.7)	(49.5–69.9)	(55.4–75.3)	(52.2–71.1)	(41.3–59.1)
**Later**	24.9	17.2	24.2	33.2	43.0
	(15.8–36.8)	(10.9–26.2)	(15.7–35.4)	(22.4–46.1)	(34.3–52.0)
**No more**	0.5	22.7	9.8	4.7	6.8
	(0.1–4.0)	(15.7–31.5)	(5.0–18.2)	(1.5–14.0)	(3.1–14.4)

The proportion of women who reported being in a union before first childbirth declined between 1995 and 2016 from 64.9 to 55.8% (− 9.1 pp., p < 0.001). This was predominantly due to a decline among rural women (66.4% in 1995 and 56.4% in 2016, − 10 pp., p < 0.001). There was no evidence of decline in the percentage of urban women who reported being in a union before first birth (54.9% in 1995 and 53.7% in 2016, *p* = 0.808).

### Determinants of adolescent childbirth (birth < 20 years) on 2016 survey

#### Women of rural residence

As shown Tables 5, 2678 women aged 20–24 years in 2016 were from rural residence and of these, 583 (21.8%) were from the Central zone. Regarding household wealth index, 879 (32.8%) of the women were in the richer and richest wealth quintiles combined. Approximately 50% of the women had complete primary education and above. The mean age at first sex was 16.6 in the sample and 15.7 years among those with adolescent childbirth. In crude Poisson analysis, compared to women from Central zone, women from Northern zone were more likely to report adolescent childbirth (crude PR 1.15, 95% CI = 1.03–1.28). Other factors crudely associated with reporting adolescent childbirth were among rural women were religion, education, household wealth, being in union before first birth, and age at first sex.

**Table 5:** Determinants of adolescent childbirth among women of rural residence, age 20–24 years at 2016 UDHS (*N* = 2678)

Variables	Total (N)	Adolescent childbirth (row%; 95% CI)	Crude PR (95% CI)	P-value (crude)	Adjusted PR (95% CI)	Wald test P-value
**Zone**						
Central	583	57.3 (52.1–62.4)	1		1	
Eastern	886	60.1 (55.7–64.3)	1.05 (0.93–1.18)	0.420	0.88 (0.80–0.96)	0.004
Northern	515	66.0 (61.9–69.8)	1.15 (1.03–1.28)	0.011	0.92 (0.84–1.00)	0.063
Western	693	59.9 (55.2–64.4)	1.04 (0.93–1.18)	0.473	0.96 (0.88–1.05)	0.416
**Religion**						
Anglican	1231	57.2 (53.6–60.6)	1		1	
Catholic	1016	62.8 (58.9–66.6)	1.10 (1.01–1.20)	0.032	1.07 (0.99–1.15)	0.062
Muslim	355	65.0 (58.2–71.3)	1.14 (1.01–1.28)	0.034	1.13 (1.02–1.26)	0.020
Other	076	64.5 (53.4–74.3)	1.13 (0.95–1.34)	0.172	1.06 (0.92–1.24)	0.410
**Education attainment**						
No and incomplete primary	1328	74.5 (71.8–77.0)	1		1	
Complete primary and higher	1350	46.9 (43.7–50.0)	0.63 (0.59–0.68)	< 0.001	0.84 (0.78–0.90)	< 0.001
**Household wealth index**						
Poorest	588	74.2 (70.5–77.5)	1		1	
Poorer	657	65.1 (60.7–69.3)	0.88 (0.81–0.95)	0.001	0.95 (0.89–1.02)	0.185
Middle	554	62.5 (58.0–66.8)	0.84 (0.77–0.92)	< 0.001	0.98 (0.91–1.07)	0.693
Richer	561	49.0 (44.1–53.9)	0.66 (0.59–0.74)	< 0.001	0.84 (0.76–0.94)	0.001
Richest	318	43.1 (37.1–49.2)	0.58 (0.50–0.67)	< 0.001	0.76 (0.66–0.88)	< 0.001
**In Union before first birth**						
No	1252	56.5 (53.2–59.7)	1		1	
Yes	1426	64.2 (61.1–67.1)	1.14 (1.06–1.22)	< 0.001	0.99 (0.94–1.05)	0.797
**Age at 1st sex***-mean years (SD)*	16.6 (2.21)	15.7 (1.75)	0.84 (0.83–0.86)	< 0.001	0.85 (0.84–0.87)	< 0.001

In multivariate analysis with all determinants, all five factors investigated remained associated with reporting adolescent childbirth. Women from Eastern zone compared to central zone (aPR 0.88, 95% CI = 0.80–0.96) and those who had completed primary and higher education compare to those with no and incomplete primary education (aPR 0.84, 95% CI = 0.78–0.90) were less likely to report adolescent childbirth. Women with household wealth index in the richer and richest categories were less likely to report the outcome. With each yearly increase in age at first sex, women were less likely to report adolescent childbirth (aPR 0.85, 95% CI = 0.84–0.87). Muslim women were more likely to report the outcome compared to Anglicans.

*NB: In calculating mean age at first sex, 50 had inconsistent values and were excluded in calculating mean age.*

*PR = Prevalence risk.*

#### Women of urban residence

As shown in Table 6, of the 1144 women age 20–24 from urban residence; 676 (59.1%) were from Central zone. Women with complete primary and higher education comprised 82.5% of this sample. Regarding household wealth index, 979 (85.6%) of the women were in the richer and richest wealth quintiles combined. The mean age at first sex was 17.3 years in the sample and 15.9 years among those with adolescent childbirth. In crude Poisson analysis, Muslim women were more likely to report adolescent childbirth (crude PR 1.29, 95% CI = 1.02–1.64) compared to Anglican women. Other factors associated with reporting adolescent childbirth among urban women among were education, household wealth, being in union before first birth, and age at first sex. Zone was not associated with the outcome in crude analysis.

**Table 6:** Determinants of adolescent childbirth among women of urban residence, age 20–24 years at 2016 UDHS (*N* = 1144)

Variables	Total (N)	Adolescent childbirth (%; 95% CI)	Crude PR (95% CI)	P-value (crude)	Adjusted PR (95% CI)	Wald test ***p***-value
**Zone**						
Central	676	36.5 (31.0–42.4)	1		1	
Eastern	160	40.6 (33.2–48.4)	1.11 (0.87–1.42)	0.401	0.93 (0.76–1.14)	0.483
Northern	098	40.2 (32.3–48.6)	1.10 (0.85–1.42)	0.465	0.93 (0.76–1.14)	0.466
Western	209	45.3 (35.0–56.1)	1.24 (0.94–1.65)	0.134	1.01 (0.77–1.31)	0.953
**Religion**						
Anglican	480	36.5 (30.2–43.3)	1		1	
Catholic	397	38.7 (33.1–44.7)	1.06 (0.83–1.35)	0.636	1.08 (0.86–1.37)	0.492
Muslim	227	47.2 (38.4–56.2)	1.29 (1.02–1.64)	0.035	1.08 (0.86–1.34)	0.518
Other	039	25.0 (11.0–47.5)	0.69 (0.33–1.42)	0.306	0.94 (0.51–1.72)	0.840
**Education attainment**						
No and Incomplete primary	200	69.2 (62.0–75.5)	1			1
Complete primary and higher	944	32.6 (28.5–37.1)	0.47 (0.40–0.56)	< 0.001	0.74 (0.61–0.89)	0.001
**Household wealth index**						
Poorest	059	59.9 (45.5–72.8)	1		1	
Poorer	044	60.0 (46.2–72.5)	1.00 (0.73–1.38)	0.987	0.88 (0.67–1.15)	0.340
Middle	062	46.8 (31.3–62.9)	0.78 (0.54–1.13)	0.189	0.83 (0.59–1.17)	0.284
Richer	177	49.5 (40.1–58.9)	0.83 (0.60–1.13)	0.234	0.84 (0.62–1.12)	0.227
Richest	802	33.4 (28.9–38.3)	0.56 (0.42–0.73)	< 0.001	0.77 (0.57–1.03)	0.077
**In union before first birth**						
No	638	32.4 (27.6–37.5)	1		1	
Yes	505	47.4 (40.8–54.1)	1.46 (1.18–1.82)	0.001	1.04 (0.84–1.28)	0.741
**Age at 1st sex -***mean years (SD)*	17.3 (2.26)	15.9 (1.80)	0.77 (0.75–0.80)	< 0.001	0.79 (0.76–0.81)	< 0.001

In multivariate analysis, only two of the factors examined, education and age at first sex, were associated with reporting adolescent childbirth in this urban sample. Women with a complete primary education and higher were less likely to report adolescent birth (aPR 0.74, 95% CI = 0.61–0.89) compared to those with no and incomplete primary education. Each additional year increase in age at first sex was associated with a lower likelihood of reporting adolescent childbirth (aPR 0.79, 95% CI = 0.76–0.81).

## Discussion

### Levels and time trends of adolescent childbirth

We found that adolescent childbirth is high; with approximately 1 in every 2 women age 20–24 reporting having had a live childbirth before the age of 20 years. The greatest burden was in rural areas; almost double the level in urban areas. In the time trends, we found a decline in adolescent childbirth in Uganda among both rural and urban women; the percentage point change was similar despite different baseline levels. The decline in adolescent childbirth started after 2000/01 and this decline predominantly affected adolescent childbirth < 18 years of age. Adolescent childbirth among girls 18–19.9 years has remained virtually unchanged over the 30 years under investigation. Over time, more and more women from both rural and urban residence wished to have had that pregnancy later. There was a decline in the percentage of rural women who were in a union before first birth.

The decline in adolescent childbirth, specifically childbirth among the younger group- < 18 years, which started after 2000/01, is encouraging and suggests that policies and programs implemented during this period, such as universal primary education and ending child marriage, might be having an effect among girls of both rural and urban residence. The decline in adolescent childbirth may also signal changes in the fabric of the society overtime such as; increase in availability of information and services for adolescents - including contraception [31], changing aspirations for the girl child, increasing school enrolment and continuation and the attitude of parents/guardians towards childbirth [32]. The constitution of Uganda prohibits both sexual intercourse and marriage of children below 18 years and Universal Primary Education was introduced in 1997 [22–24]. Women 18 years and above are not prohibited and have a right to marriage and childbirth. These policies were implemented gradually and if effective, they would have started to affect adolescent childbirth patterns in the early 2000s. This coincides with our finding of the timing of the change, a decline in adolescent birth including proportions who wanted the pregnancy then and those in union before first birth, but we are not able to make a causal inference due to the cross-sectional nature of the data. Uganda has registered an increase in the enrolment of girls in school [33–35] and a reduction in early marriage in the last 15 years [16]. Other studies in Uganda have demonstrated a decline in adolescent childbearing but did not indicate when the decline set in [36–38]. Using the DHS data for Uganda, Kenya and Tanzania, Neal et al. (2015) concluded that there was a very slight reduction in proportion of women reporting first adolescent birth in East Africa [37]. Based on the analysis of the Uganda DHS 2011, Kenya DHS 2008/9, and Tanzania DHS 2010, the proportions of women age 20–24 years reporting adolescent childbirth in Kenya and Tanzania were; 47 and 56% respectively, at the time of the survey. The authors analysed the UDHS of 1988/89 and 2011 and concluded that there was a very slight progress in reduction of adolescent childbirths among < 18-year olds, although they did not investigate when these reductions occurred. Sub-Saharan African countries that have consistently applied their laws of minimum marriage age at 18 years or older are reported to have registered a decline in adolescent childbearing [21].

Our results are supported by earlier findings of high adolescent fertility in the midst of low contraception access and uptake, high unmet need, and no formal comprehensive sexuality education in schools in Uganda and East Africa [12, 20, 31, 39–41]. When adolescents access contraception, they usually use methods that require regular action from the user for effectiveness [42]. In Uganda, the methods most commonly used by the few adolescents that use are; condoms, injectable (3 monthly), one month pill packs and emergency contraception pills [41]. The greater burden of adolescent childbirth among the rural women might be due to differences these opportunities such as; access to health information, education opportunities, economic activities, and family protection [25–27, 43, 44].

Progress in reducing adolescent childbirth has been uneven globally with variations within and across countries [45]. Progress has been much better in higher income countries than sub-Sahara Africa with variations across countries within the regions [20, 21, 46, 47]. Whereas there has been a plateau in West Africa, East Africa registered a decline in adolescent pregnancy between 1992 to 2011 [20]. In the United States of America and many European countries, the last three decades have registered further decline in their already lower adolescent childbirth rates [10, 48–50]. The declines in the United States were attributed mainly to improved contraceptive use among others. A study by Lindberg et al. (2016), using the United States data from the National Surveys of Family Growth for young women (15–19 years) in the periods 2007, 2009 and 2012, found that pregnancy risk index declined at an annual rate of 5.6% (*p* = 0.071) from 2007 to 2012 due to improved contraception use [51]. The studies acknowledge that beyond the program efforts, the decline may be due to the changing aspirations of the girls such as, a greater number aspiring for greater educational and career achievements [49, 51]. These findings, in other settings, highlight the need for Uganda to improve contraception services and other opportunities for adolescent women, if further reductions in adolescent childbirth are to be realized.

### Determinants of adolescent childbirth

In our study, the determinants associated with reporting adolescent childbirth (birth < 20 years) among both rural and urban women were: no or incomplete primary education and younger age at first sex. Among women from the rural residence, the additional risk factors for adolescent birth were: being from Eastern zone, high household poverty, and Muslim religion. Urban residents had only the two factors; no or incomplete primary education and younger age at first sex. These results reinforce the role education, poverty and early age of sexual debut in increasing the risk of adolescent childbirth/pregnancy [2, 25, 52–55]. Girls from poorer communities and households are more vulnerable to adolescent pregnancy and early marriage/union. The most vulnerable to these challenges are those from rural settings. Poverty is associated with poor access to educational opportunities, health information, decision making regarding contraception use, sexual coercion and abuse, among others, and vice versa. Low or no education is associated with reverse causality; leads to poverty and inability to access other opportunities. Studies in Uganda and other LMICs found that girls from poor households and communities are more vulnerable to pregnancy, discontinuation of education, sexual coercion and abuse, and early marriage/union [20, 25, 56]. Any form of union is a risk factor for childbirth as it directly increases the exposure of the girl to pregnancy, societal or personal pressures to start a family, and it diminishes the adolescent girl’s ability to make her own choices. Irrespective of marital status, the earlier the girl starts sex, the more vulnerable she is to engage in high risk sex with a high probability of not using contraception. Studies in high income countries have also shown that adolescent girls form poorer communities are at higher risk for pregnancy/childbirth [52, 57].

Muslim religion was significantly associated with adolescent childbirth especially among the rural residents. Religious affiliation or the lack of it has been linked to influence adolescent fertility although there are conflicting findings, with some reporting no influence [58, 59]. The Muslim faith promotes early marriage [21] which is a risk factor for adolescent pregnancy. In Uganda, Muslim girls were found to be at higher risk of pregnancy than the protestant girls [37]. Other studies in sub-Sahara Africa have found higher fertility among Muslim adolescents or in regions with predominantly Muslim religion [60, 61]. A study in the Netherlands found pregnancy more common among Muslim girls than other minority groups [62].

The geographical place of living (region) has been shown to influence adolescent pregnancy risk due to possibly variations in opportunities and social norms, among others [25]. A previous DHS analysis showed variations in adolescent childbirths by regions in Uganda, Kenya and Tanzania [39]. In our study, age at first sex was different among urban and rural women. This difference may have influenced the fertility patterns observed from the different regions of the country that are in contrast to previous reports in Uganda that indicated the Eastern and Northern regions have higher proportions of adolescent girls who begun childbearing [63].

### Limitations

The respondents gave self-reported information for which we cannot ascertain the accuracy. Recall bias is an inherent problem as women are asked about events up to five years prior to the survey and in certain instances, women may potentially misreport information for sensitive topics such as age at first sex [64]. The DHS are cross sectional in nature, only associations can be provided and not causality. This is because what is being assessed as a determinant at the point of survey may have been a result or consequence of the outcome of interest. We cannot, based on our results, conclusively say that the government programs and legislation led to the reduction in adolescent childbirth because many factors, such as changes in societal norms or possible increase in prevalence of abortions, may have played a role. Further, we might have slightly underestimated the percentage of adolescent childbirth because using live births – do not account for pregnancies that did not result in live birth, as there was no data on miscarriages, abortions and stillbirths. The proportions of births in the year 1988/89 and 2000/01 might also have been affected by the exclusion of some districts. While we cannot exclude residual confounding, we are confident that we included the important variables to have an impact on adolescent childbearing. The urban/rural categories refer to the point of interview rather than during adolescence and yet this could have been affected by rural-urban migration in early adulthood [65] and perhaps after childbirth. However, this rural-urban distribution among adolescents 15–19 years is similar with 77% being from rural residence as per the 2014 National Population Census [66] therefore, we believe there may not have been bias. The category of women age 20–24 years carries a longer time lag, 2.5–5 years, in information than the 15–19-year old who would perhaps provide more current information of events during adolescence. This time lag may potentially coincide with other changes in the society at that time and therefore not speak into the current. Nonetheless, this DHS data avail an opportunity, in the absence of prospective data, to track what has happened in the past and thereby improve programming.

## Conclusions

Adolescent childbirth among both rural and urban women, although high in Uganda, has declined over the last 30 years with more wanting to delay the pregnancy and fewer reproitng being in union before first birth. The overall decline was due to reduction in first childbirth among the younger age group, girls < 18 years but not in 18–19 years old. To further reduce adolescent childbearing, efforts need to be intensified to keep girls in school, alleviate household poverty and delay age at first sex among girls of both urban and rural residence. Provision of scientific sexuality education and improving access to modern contraceptives for adolescents needs to be fast tracked. In addition, strategies should factor in the differences in risk by religion as Muslim girls are at high risk of adolescent childbirth.

Studies are needed to understand which interventions, if any, are contributing to the decline in adolescent childbirth among women < 18 years of age, harvest best practices and scale them up. There is need to study which determinants, if any, may have changed over time and potentially contributed to the decline. Further, we recommend that future research investigate contraception use as a potential determinant.

## Data Availability

The datasets analysed for the study are available in the DHS program website. This is accessible by non-academics and available at https://www.dhsprogram.com/.

## Abbreviations

DHS: Demographic Health Survey
LMIC: Low- Middle-Income Country
SOMREC: School of Medicine Regulatory Ethics Council
UDHS: Uganda Demographic Health Survey
UNCST: Uganda National Council for Science and Technology
UPE: Universal Primary Education
USE: Universal Secondary Education
